# Bactericidal and cell membrane disruption potential of protein extract from buttermilk-derived probiotic *Pediococcus pentosaceus* OBK05 against *Salmonella typhimurium* ATCC 14028

**DOI:** 10.3389/fmicb.2026.1869302

**Published:** 2026-08-05

**Authors:** Priyadarshini Boda, Mohammad Saddam Hussain, Bhima Bhukya

**Affiliations:** Centre for Microbial and Fermentation Technology, Department of Microbiology, University College of Science, Osmania University, Hyderabad, Telangana, India

**Keywords:** antimicrobial proteins, foodborne pathogen, probiotics, *Salmonella typhimurium*, SEM

## Abstract

**Introduction:**

Foodborne Salmonellosis remains a significant global health challenge, and the rise of antimicrobial resistance is limiting the efficacy of traditional antibiotic treatments, emphasising the need to develop natural biopreservatives to improve food safety. The present study evaluates the antimicrobial potential of a bioactive protein fraction isolated from the probiotic isolate *Pediococcus pentosaceus* OBK05 against the predominant foodborne pathogen, *Salmonella typhimurium* ATCC 14028.

**Methods:**

Antimicrobial protein was extracted from *Pediococcus pentosaceus* OBK05 and its inhibitory properties were evaluated through a series of standardised *in vitro* assays including growth kinetics monitoring and membrane disruptive effects were determined by cell membrane integrity assay and bacterial ultrastructure was determined by Scanning electron microscope.

**Results:**

The protein extract showed dose-dependent reduction in pathogen growth with the minimum inhibitory concentration. Growth kinetics and viable cell count revealed a rapid bactericidal effect of the protein extract. The mode of action of the extract involved changes in the membrane structure of pathogen cells, causing metabolic disruption and cellular homeostatic imbalance, as evidenced by the progressive release of intracellular molecules. Furthermore, alterations in cellular structure and function induced by the protein extract were investigated using scanning electron microscopy and analysis of protein expression patterns in treated *Salmonella* cells.

**Discussion:**

Together, these findings demonstrate a multitargeted antimicrobial mechanism of the bioactive protein fraction from *Pediococcus pentosaceus* OBK05 against *Salmonella typhimurium* ATCC 14028, establishing its potential as an effective natural biocontrol agent to enhance food safety.

## Graphical abstract

**Figure:**
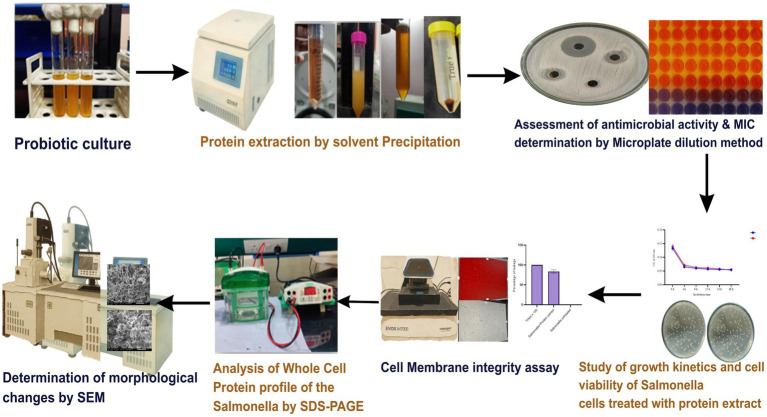

## 1 Introduction

Foodborne diseases are one of the main causes of morbidity and mortality in low-income communities, where sanitation and hygiene levels are extremely poor. On the other hand, poverty and improper food storage facilities are other contributing factors (Fung et al., 2018). Unsafe food is a threat to human health and the economy, disproportionately affecting vulnerable and marginalised people, especially women and children, as evidenced by about 600 million cases of foodborne diseases reported annually across the world (World Health Organization, 2023). Major foodborne pathogens and food spoilage microorganisms include the genera *Salmonella, Escherichia, Pseudomonas, Shigella, Klebsiella, Proteus, Bacillus*, and *Listeria* (Bintsis, 2017). Among these pathogens, *Salmonella* is a significant global health concern due to its widespread transmission via contaminated food and water, leading to gastrointestinal infections in humans (Lu et al., 2025). Approximately 95 million cases of Salmonella infection were reported globally each year, with an estimated 120,000 deaths (Kungu et al., 2026). Salmonella infection in humans varies in severity by serotype and host immunity, and is categorised into non-typhoidal and typhoidal forms. Non-typhoidal Salmonella infections are characterised by an acute onset of gastrointestinal illness with diarrhoea and abdominal discomfort; in contrast, typhoidal serovars produce generalised systemic infections (Lamichhane et al., 2024). The treatment of these infections relies on antibiotic susceptibility testing; the emergence of multidrug-resistant strains linked to antibiotic misuse has complicated therapeutic outcomes and potentially altered the composition and impact of the gastrointestinal tract flora (Patangia et al., 2022). Historically, chemical preservatives and antibiotics have controlled these infections, but antimicrobial resistance and consumer demand for eco-friendly, residue-free food have galvanised the search for alternative approaches (Helmy et al., 2023). To restore microbial equilibrium and prevent disease, the gastrointestinal tract may benefit from the introduction of beneficial bacterial species and their metabolites, particularly antimicrobial peptides, which have garnered considerable research interest (Jiao et al., 2025). The possible role of probiotics in preventing or reducing the risk of foodborne infections has been the subject of research (Amalaradjou and Bhunia, 2012). The efficacy of probiotics is linked to their ability to regulate the immune system, modulate the gut microbial community (Latif et al., 2023), and synthesise bioactive compounds produced during fermentation or protein hydrolysis (Mazziotta et al., 2023). Among these bioactive compounds, antimicrobial proteins produced by probiotic bacteria have emerged as potential alternatives to conventional antibiotics and synthetic food preservatives (Niknam et al., 2025). These probiotics employ multifaceted mechanisms, including bacteriocin production and direct protection of the gut mucosa (Wang et al., 2021). Bacteriocin is a generic term used for peptides with antimicrobial activity. They are produced as adaptations that allow probiotic bacteria to compete with other microbes. The mode of action of bacteriocins is by cell wall disruption via hydrolysis of peptide or glycosidic bonds. This target mechanism confers broad-spectrum antimicrobial activity, acting against both Gram-positive and Gram-negative bacteria. Considerable attention has been directed to use these antimicrobial peptides as biocontrol agents in the food industry. Therefore, identification and characterisation of novel antimicrobial peptides remain an important area of research, and for this, different fermented and non-fermented sources are extensively explored lately (García-Cano et al., 2019).

Despite the growing popularity of dairy-based probiotics, antimicrobial peptide-producing strains from buttermilk have received relatively less attention than those from other fermented dairy products like whey. This highlights that traditionally fermented buttermilk is a potential source of novel probiotic bacteria. Buttermilk is similar to whey in lactose content but contains higher amounts of proteins and bioactive compounds like butyrophilin, adipophilin, phospholipids, sphingolipids, mucin-like glycoproteins, breast cancer type 1 and 2 sensitivity proteins, etc. Traditionally fermented buttermilk has an excellent environment for probiotic bacterial growth, as it contains lactose and other nutrients that act as prebiotics (Elkashef et al., 2026). In traditionally fermented buttermilk, the microbial consortium in buttermilk enhances the nutritional profile and ensures safety for consumption by producing bioactive components, including short-chain fatty acids, amino acids, vitamins, and enzymes (İncili et al., 2023). Fermented buttermilk improves lactose digestion, manages cholesterol and shows immunomodulatory effects mainly attributed by its microbiota. These microbes are involved in selective inhibition of many food-borne pathogens as they produce range of antimicrobial compounds like bacteriocins, reuterin, hydrogen peroxide, D-isomers of amino acids, diacetyl, acetaldehyde; etc. (Atteya et al., 2025). Antimicrobial peptides are used as a first-line barrier against microbial attack, thereby acting as biopreservatives in food applications. *Lc. lactis*, *Pc. pentosaceus*, *Pc. acidilactici,* and *Lb. delbrueckii* are reported to produce bioactive compounds including antimicrobial peptides, which are released in cell-free supernatants (Hati et al., 2018). The antimicrobials produced by *Pediococcus* sp. are also reported to exhibit antagonistic activity against many foodborne pathogens, and interestingly, can inhibit *Listeria monocytogenes* growing at temperatures as low as 2–4 °C. This demonstrates its potential as a promising bio-preservative agent (Amenu and Bacha, 2025).

The aim of the present study was to extract antimicrobial proteins from the selected antagonistic probiotic isolate *Pediococcus pentosaceus* OBK05 originally isolated from traditionally fermented buttermilk, and evaluate its inhibitory potential against *Salmonella* spp. Additionally, the study investigated the underlying antimicrobial mechanism with bacterial membrane integrity, permeability, and cellular morphology as key factors. Overall, the study aimed to elucidate the antimicrobial efficacy of the probiotic protein extract from a select probiotic isolate and to understand the molecular mechanisms underlying its interactions with *Salmonella typhimurium* ATCC 14028. The study specifically assessed its effect on membrane integrity and cellular morphology as an efficient alternative biotherapeutic agent. The study also demonstrates the feasibility of using these antimicrobial proteins as sustainable alternatives to antibiotics, thereby contributing to the development of novel biopreservation strategies in this era of increasing antimicrobial resistance.

## 2 Materials and methods

### 2.1 Bacterial cultures and growth conditions

The probiotic bacterial isolate used in this study, *Pediococcus pentosaceus* OBK05, was selected based on its reported antimicrobial potential and previously established probiotic profiles, along with the standard reference strain, *Lactobacillus acidophilus* NCIM 2285. This strain was initially isolated from buttermilk, and bacterial identification was performed using 16S rRNA sequencing, as described in our previous work (Bhukya and Bhukya, 2021). This bacterium was used to extract antimicrobial proteins (AMPs) because of its demonstrated inhibitory activity against clinical pathogens. The isolate was retrieved from 20% glycerol stocks maintained at −80 °C and cultivated in deMan, rogosa, and Sharpe (MRS, Hi-Media, India) medium at 37 °C for 24 to 48 h. To study the antimicrobial properties of the extracted proteins, various foodborne pathogens were used as indicator organisms, like *Staphylococcus aureus* (ATCC 43300), *Bacillus cereus* (ATCC 14579), *Enterococcus faecalis* (ATCC 29212), *Escherichia coli* (ATCC 25922), *Salmonella enterica serovar typhimurium* (*S. typhimurium* ATCC 14028*), Klebsiella pneumoniae* (ATCC 700603), *Proteus mirabilis* (ATCC 25933), and *Streptococcus mutans* (ATCC 700610), which were procured from the American Type Culture Collection. All the above pathogenic bacteria were grown in Brain Heart infusion (BHI) medium (Hi-Media, India) under constant shaking at 37 °C.

### 2.2 Extraction of antimicrobial proteins from probiotic isolates

The Probiotic bacterial culture was grown in 100 mL MRS broth at 37 °C for 24 h to reach the late exponential phase. The cell-free supernatant (CFS) was obtained by centrifugation at 6000 × *g* for 10 min at 4 °C. The resulting supernatant was neutralised to pH 7.0 using KOH and then passed through a 0.45-μm pore-size filter (Millipore) to ensure complete removal of cellular debris, maximise protein recovery and preserve antimicrobial activity. Crude proteins were extracted from the CFS using a modified TCA-acetone method (Alam and Ghosh, 2014). Briefly, four parts (400 mL) of ice cold 15% (v/v) TCA-acetone were added to the CFS (100 mL) in a sterile (50 mL) vial, and the mixture was vortexed gently and incubated overnight at −20 °C to facilitate protein precipitation. Following incubation, the mixture was centrifuged at 10,000 rpm for 10 min at 4 °C, and the resulting protein pellet was collected and washed with ice-cold acetone (HPLC grade, ≥99.5%). After air drying to remove residual solvent, the pellets were resuspended in phosphate-buffered saline (PBS, pH 7.8). Finally, the peptide concentration in the crude extract was quantified using the Bradford method (Kruger, 2009). The protein content of uninoculated sterile MRS broth was subtracted from the protein content of inoculated sample values to determine the net antimicrobial protein concentration produced specifically by the probiotic isolate.

### 2.3 Protease and catalase sensitivity assays of protein extract

To demonstrate that the observed antimicrobial activity was attributable to proteins or peptides rather than to residual organic acids, hydrogen peroxide or small-molecule metabolites, the crude protein extract was incubated with catalase (100 U/mL) for 2 h at 37 °C, and heat treated at 100 °C for 10 min to inhibit enzyme activity (Lim et al., 2018). The protein nature of the extract was assessed by the modified method reported earlier by Bindu and Lakshmidevi (2021) where the crude protein extract was subjected to Proteinase K and Pepsin enzyme hydrolysis. The protein extract with Proteinase K and Pepsin (HiMedia, Mumbai, India) enzymes at a final concentration of 1 mg/mL each in their respective optimal buffers, 10 mM Tris HCl (pH 7.5) and 0.002 M HCl (pH 2.0), respectively, were incubated at 37 °C for 30 min. After incubation, the enzyme activity was terminated by heating the mixture in a boiling water bath for 5 min. The extract was subjected to molecular weight-based fractionation using ultrafiltration with 3.5 KDa molecular weight cutoff (MWCO) filters. Additionally, to confirm the removal of TCA carryover from the extraction process (Naveed et al., 2023), the extract was transferred to Amicon ultra 3.5 KDa MWCO centrifugal filter units (Millipore, USA) and centrifuged at 10000 × *g* at 4 °C until 10–15% of the original volume remained in the filter unit. The retentate (>10 KDa) and filtrate (<10 KDa) were collected separately. The antimicrobial activities of both the enzyme-treated extracts along with the fractions obtained by ultrafiltration were then tested against test pathogen *Salmonella typhimurium ATCC* 14028 by agar well diffusion assay. Untreated extract and the enzymes in their respective buffers served as controls.

### 2.4 Assessment of antimicrobial activity of the probiotic protein extract

The antimicrobial potential of PPE from *Pediococcus pentosaceus* OBK05 and *Lactobacillus acidophilus* NCIM 2285 was determined using the agar well diffusion assay with fewer modifications (Bhukya and Bhukya, 2021). Indicator pathogenic strains (previously described in section 2.1) were cultured in BHI broth overnight at 37 °C with shaking (150 rpm), and cultures were standardised with sterile saline to a turbidity of 0.5 McFarland (approximately 1.5 × 10^8^ CFU/mL). The standardised cell suspensions were then uniformly inoculated on nutrient agar plates using sterile cotton swabs. Wells 6 mm in diameter were made using a sterile cork-borer, and each well was loaded with 80 μL of the respective protein extract with Nisin (Sigma-Aldrich, USA) 50 μg/mL as a positive control. The plates were incubated at 37 °C for 24 h, after which the antimicrobial activity was determined by measuring the diameter of the zone of inhibition around each well using a ruler. All assays were performed in triplicate to ensure statistical reliability.

### 2.5 Minimum inhibitory concentration of PPE

The minimum inhibitory concentration of protein extracts was determined using the broth microdilution method in a 96-well microtiter plate (Wiegand et al., 2008). The initial concentrations of protein extract and nisin were set to 1,000 μg/mL, a two-fold serial dilution was performed in Mueller-Hinton broth medium to achieve a concentration range of 1,000–125 μg/mL. Each well was subsequently inoculated with active pathogenic cultures (prepared as described in section 2.3) standardised to a final concentration of 10^5^ CFU/mL. The plates were incubated at 37 °C for 24 h. Post-incubation, microbial growth was quantified by measuring the OD_600_ on a multimode microplate reader (Thermo Scientific). The minimum inhibitory concentration (MIC) was determined as the protein concentration (μg/mL) that completely inhibited visible growth, as evidenced by no increase in OD_600_ compared to the negative control sterile MRS broth. To ensure assay validity, phosphate-buffered saline (pH 7.0) was used as a negative control, and 15% (v/v) TCA-acetone was used as a solvent control. The minimum bactericidal concentration (MBC) of the extract was determined by inoculating 10 μL culture aliquots from wells onto sterile nutrient agar plates, which were then incubated at 37 °C for 24 h. The MBC was defined as the lowest concentration that completely inhibited bacterial growth, as evidenced by the absence of growth on the plates.

### 2.6 Safety evaluation of PPE

#### 2.6.1 Haemolysis assay

The haemolysis assay was adapted from previous studies to evaluate the safety features of protein extract (Ebbensgaard et al., 2018). Fresh defibrinated sheep blood was washed thrice with PBS (pH 7.0) by centrifugation for 15 min each at 1000 × *g*, and resulting erythrocytes (10%) were resuspended in PBS. A 100 μL volume of erythrocyte suspension was mixed with protein extract at different concentrations. A minimum protein extract concentration of 50 μg/mL and a maximum of 400 μg/mL were selected for the assay. Triton x-100 (0.2%) and PBS were considered as positive and negative controls, respectively. Following a 60 min incubation, samples were centrifuged for 10 min at 1300 × *g* to pellet intact erythrocytes and remove cellular debris. The resulting supernatant was transferred to a 96 well microtiter plate, and the haemoglobin release was observed by measuring the absorbance at 540 nm on a multimode microplate reader (Thermo Scientific). The percentage of haemolysis was calculated as Percentage of haemolysis = (A_s_ − A_b_)/(A_c_ –A_b_) x 100, where A_s_ is the absorbance of extract- treated cells, A_b_ is the absorbance of untreated erythrocytes (positive control), and A_c_ is the absorbance of 0.2% Triton x-100 lysed cells.

#### 2.6.2 Cytotoxicity assay

Human embryonic kidney cell line (HEK293) sourced from National Centre for Cell Science, Pune, India was taken as a normal cell line to assess the cell toxicity effect of protein extract (Popiołkiewicz et al., 2005). Cells were maintained in high glucose DMEM medium enriched with 10% foetal bovine serum, 1% penicillin–streptomycin antibiotic, 2 mM L-glutamine and 0.37% sodium carbonate. Once cells reached 80% confluency as a monolayer, they were enzymatically detached using trypsin–EDTA solution, then rinsed twice with complete medium at a seeding density of 1 × 10^4 cells per well. The extract was treated at different concentrations and incubated with cells for 24 h at 37 °C in a 5% CO_2_ atmosphere. Following incubation, the culture medium was removed, and the cells were washed with PBS; the cells were then treated with MTT dye (0.5 mg/mL) and incubated for 4 h. The formazan crystals were then solubilised by adding 200 μL of DMSO, and the resulting colour intensity was quantified at 570 nm using a multimode microplate reader. Cell viability was calculated by comparing extract-treated cells to untreated control cells, with DMSO-treated wells serving as a blank reference. Per cent cell viability = (A_t_ – A_b_/A_c_-A_b_) x 100, where A_t_ is absorbance of extract-treated cells, A_b_ is absorbance of blank (DMSO), and A_c_ is absorbance of control (untreated cells).

### 2.7 Stability studies of PPE

The thermal and pH stabilities of protein extract were evaluated by incubating the extract for 30 min at various temperatures (4, 20, 40, 60, 80, 100 and 121 °C) and incubating in buffers with different pH values ranging from 2, 4, 6, 8, 10 (Martinez et al., 2013). Following incubation, residual antimicrobial activity of treated extract samples was assessed against the test pathogen, *Salmonella typhimurium*, using a sterile 96-well microtiter plate method, in which pathogen-seeded plates were treated with protein extract samples and incubated for 24 h at 37 °C. The growth rate of the pathogen strain was measured by the turbidimetric method using a multimode microplate reader (Thermo Scientific) at OD_600_. The results were estimated as the percentage inhibition by the treated extract and calculated as (At – Ab)/(Ac – Ab) × 100, where At is the absorbance of the test, Ab is the absorbance of the blank, and A_c_ is the absorbance of the control.

### 2.8 Effect of PPE on salmonella

#### 2.8.1 Growth kinetics

The antimicrobial effect of probiotic protein extract against *Salmonella* was evaluated by time-kill analysis following a modified method of Mazumdar et al. (2020). The pathogenic culture in the logarithmic growth phase was inoculated into sterile LB broth at a 1:100 (v/v) ratio to achieve the standard initial cell density. Protein extract was added to the bacterial suspensions at a concentration of 500 μg/mL, and the mixtures were incubated at 37 °C constant orbital shaking at 150 rpm. To monitor growth kinetics, 100 μL aliquots were withdrawn at specific time intervals: 0 h, 3 h, 6 h, 12 h, 24 h and 48 h. The optical density at 600 nm (OD_600_) was measured using a multimode microplate reader (Thermo Scientific). The viability of the bacterial cells at 0 h, 3 h, 6 h, 12 h, 24 h and 48 h was confirmed by the spread plate method on nutrient agar and the plates were incubated at 37 °C for 24 h. The resulting colony-forming units (CFU/mL) were recorded to assess the bactericidal activity of the protein extracts.

#### 2.8.2 Cell membrane integrity

The leakage of intracellular components was quantified to assess the membrane damage, following the modified method described by Han et al. (2024). Overnight-grown cultures of *Salmonella* in BHI broth (18 h, 37 °C, 150 rpm) were harvested and washed twice with 1 x phosphate-buffered saline (pH 7.5). The bacterial cells were then resuspended and treated with protein extract at a final concentration of 500 μg/mL. All the samples were incubated at 37 °C for 24 h. Following incubation, the suspensions were centrifuged at 8000 rpm for 10 min to remove the cells; the resulting supernatants were carefully collected, and the concentration of released nucleic acids was determined by measuring absorbance at 260 nm (A_260_) on a multimode microplate reader (Thermo Scientific).

##### 2.8.2.1 Propidium iodide staining

Cell membrane disintegration was further assessed by a PI uptake assay, as described by Devi et al. (2024), with some modifications to the methodology. Overnight cultures of *Salmonella* grown to log phase were harvested by centrifugation, and the cells were washed twice with PBS (pH 7.5) to remove residual growth media. The bacterial cells were standardised by resuspending in PBS and treated with protein extract at 500 μg/mL concentration and incubated at 37 °C for different time intervals up to 6 h. PI was added to bacterial cells at a concentration of 10 μg/mL and incubated in dark for 10 min at room temperature, where it binds with DNA or RNA in permeabilized cells and produces red fluorescence when observed under fluorescent microscope. A volume of 200 μL PI-stained bacterial cell suspension was added to the glass bottomed imaging chamber of Evos live cell imaging inverted fluorescent microscope and imaged at a magnification of 40x in bright field and red fluorescent channels (excitation 535 nm; emission 617 nm) to visualise morphology and membrane integrity status. Bright field and PI-fluorescence images were captured for treated bacterial cells along with controls.

#### 2.8.3 Protein expression by SDS-PAGE

The effect of PPE on the total protein of *Salmonella* was analysed using SDS-PAGE as described by Tenea (2020). The indicator pathogen was treated with protein extract at 500 μg/mL for 24 h at 37 °C, and the cells were harvested by centrifuging at 3000 rpm for 5 min. The resulting cell pellets were washed, and resuspended in 1x SDS-PAGE sample loading buffer. To facilitate protein denaturation, the samples were boiled at 100 °C for 5 min. After heating, the samples were centrifuged at 300 rpm to remove insoluble debris. The supernatants containing solubilised whole cell proteins from both treated and untreated cells were loaded onto 12% polyacrylamide gel and separated by electrophoresis using Bio-Rad Mini-PROTEAN® Tetra Cell at constant voltage 120 V for 90 min. Protein bands were subsequently visualised using Coomassie brilliant blue staining (MB092-5G, Hi-Media) to identify any degradation or alterations in the proteins. Protein ladder of 10–250 KDa range was used as molecular weight standard.

#### 2.8.4 Cell morphology

The morphological changes and ultrastructural alterations induced in *Salmonella* by probiotic protein extracts was visualised and quantitatively analysed using scanning electron microscope (SEM). Sample preparation followed a modified version of established protocol (Wen et al., 2016). In brief, overnight culture of untreated and protein extract treated *Salmonella* cells were harvested by centrifugation at 3000 × *g* for 5 min. Cell pellets were immediately processed for SEM analysis to avoid artificial artefacts. After incubation, the cells were fixed with 2.5% (v/v) cold glutaraldehyde in 0.2 M Sodium cacodylate buffer (pH 7.2) in the fixative solution at 4 °C overnight. The fixed samples were washed three times with working buffer (0.1 M SCB) with 30 min interval to remove excess fixative solution. After final dehydration, cells were treated with ethanol in ascending order (100%) from 30, 50, 70, 80, 90 and 100% at 4 °C for 1 h. The samples were removed from ethanol and air dried under high vaccum (10–7 Torr) at room temperature (25 °C) and mounted on aluminium stubs. The specimens were then coated with gold (300 A^0^) in sputter coating unit model: E-1010 Hitachi Japan) and examined under a Scanning Electron Microscope (S3400NHitachi Japan) at 10KV and scanned images were taken in different magnifications. Quantitative morphological analysis of the SEM monographs was conducted using ImageJ software (1.54 g; National institute of Health, USA) in which, for each parameter at least 25 bacterial cells were analysed from images captured at 10000 magnification. Morphological parameters including cell membrane rupture and cell surface texture uniformity were assessed. Region of interest (ROIs) were randomly selected from bacterial cell surfaces from both control and treated groups. The surface uniformity was determined by calculating standard deviation of pixel intensity (grey scale values) within each ROI. Structural damage including membrane breakage or pore formation was assessed by randomly selecting three independent SEM monographs and systematically analysed all the visible bacterial cells at constant magnification. Mean percentage rupture was calculated across all three replicates for each treatment group by averaging the individual percentages. Statistical significance was determined using a two-tailed, two-sample t-test with equal variance parameters.

### 2.9 Statistical analysis

All experiments were carried out in triplicate as independent biological replicates (*n* = 3) to ensure precision. Data were reported as mean ± standard deviation. For statistical analysis, Microsoft Office Excel 2021 (version 2,510) and GraphPad Prism (version 5.04, San Diego, CA, USA) were used. One way analysis of variance (ANOVA) was used to evaluate difference between multiple groups, *p* value of <0.05 was considered statistically significant. Non-linear regression analysis was performed using 4 parameter logistic regression model. The goodness of fit was determined by *R*^2^ value and 95% confidence intervals were calculated to assess the precision of the estimated parameters.

## 3 Results

### 3.1 Antimicrobial spectrum of the protein extracts of OBK05

**Figure 1:**
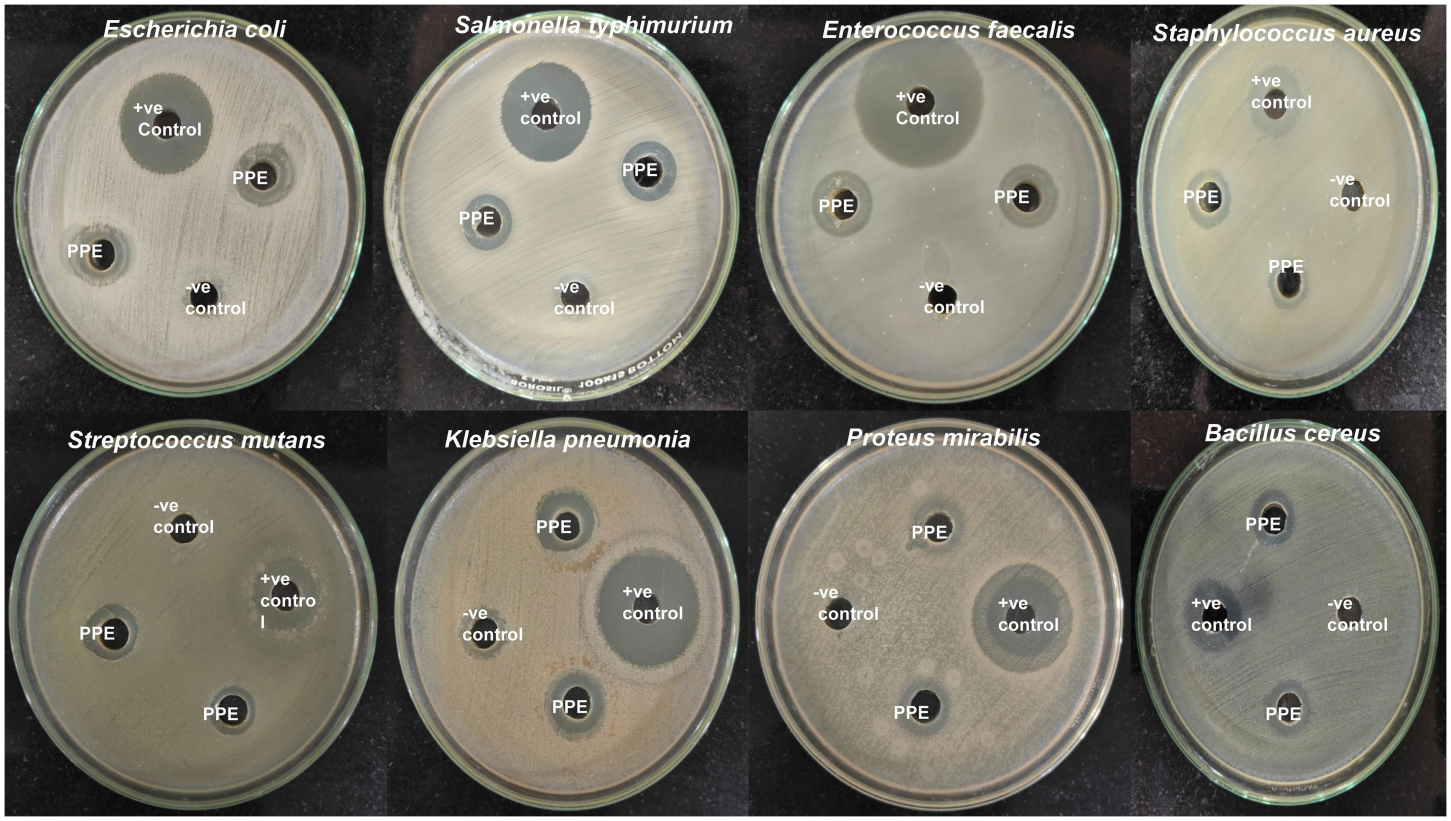
Antimicrobial activity of the protein extracts from *Pediococcus pentosaceus OBK05* and *L. acidophilus* shown as zones of inhibition against test pathogens. Experiments were carried out in triplicate, and data represent the mean ± SD (*n* = 3).

The protein extract exhibited varying degrees of antimicrobial activity against selected foodborne pathogens Formation of clear zones around the wells confirmed the potential bioactivity of extract. Among all, *Salmonella typhimurium* was found to be more susceptible to extract showing larger zone of inhibition followed by *Escherichia coli* (Table 1; Figure 1). A total protein concentration of 2.79 mg/mL was obtained using TCA-acetone precipitation.

**Table 1:** Antimicrobial activity of probiotic protein extract (PPE) from *Pediococcus pentosaceus* OBK05 and *Lactobacillus acidophilus* against a panel of foodborne pathogens determined by Zone of inhibition and Minimum inhibitory concentration.

Pathogens	Zone of inhibition (mm)			Minimum inhibitory concentration of OBK05 PPE (mg/mL)		
	PPE of OBK05	PPE of *L. acidophilus*	Nisin (Control)	MIC (μg/mL)	MIC 50 (mg/mL)	MBC (μg/mL)
*Escherichia coli* (ATCC 25922)	15.8 ± 0.15	12.64 ± 0.92	17.02 ± 0.21	500	0.2570	0.25
*Klebsiella pneumonia* (ATCC 700603)	14.9 ± 0.14	13.69 ± 0.62	24.6 ± 0.51	1,000	0.3098	1
*Staphylococcus aureus* (ATCC25923)	14.9 ± 0.18	14.61 ± 0.32	17.7 ± 0.31	1,000	0.3150	>1
*Salmonella typhimurium* (ATCC 14028)	22.9 ± 0.13	20.5 ± 0.51	24.6 ± 0.50	500	0.2701	0.5
*Bacillus cereus* (ATCC 23857)	14.9 ± 0.11	12.7 ± 0.21	16.54 ± 0.50	1,000	0.2996	>1
*Streptococcus mutans* (ATCC 700610)	16.06 ± 0.30	13.6 ± 0.31	17.6 ± 0.21	1,000	0.3570	1
*Enterococcus faecalis* (ATCC 29212)	23.1 ± 1.42	23.9 ± 1.10	26.42 ± 0.52	1,000	0.3611	1
*Proteus mirabilis* (ATCC 25933)	13.4 ± 0.61	12.7 ± 0.21	15.03 ± 0.12	1,000	0.3082	>1

### 3.2 Determination of protein nature of the crude extract

The crude extract was further assessed for its chemical nature to determine whether the protein or other components are responsible for antimicrobial activity. Systematic control experiments were conducted to confirm the activity was due to proteins but not by any non-protein metabolites. It was observed that there was no change in antimicrobial potency after catalase treatment, which confirms that H_2_O_2_ was not responsible for inhibitory effect. On the other hand, treatment of extract with protease showed loss of inhibitory activity further confirming its proteinaceous nature. Ultra filtration fractionation showed that the retentate fraction retained >90% original inhibitory activity whereas, filtrate fraction showed very less to no activity demonstrating that low molecular weight metabolites were responsible for the observed antimicrobial effects (Supplementary Figure S1).

### 3.3 Minimum inhibitory concentration

**Figure 2:**
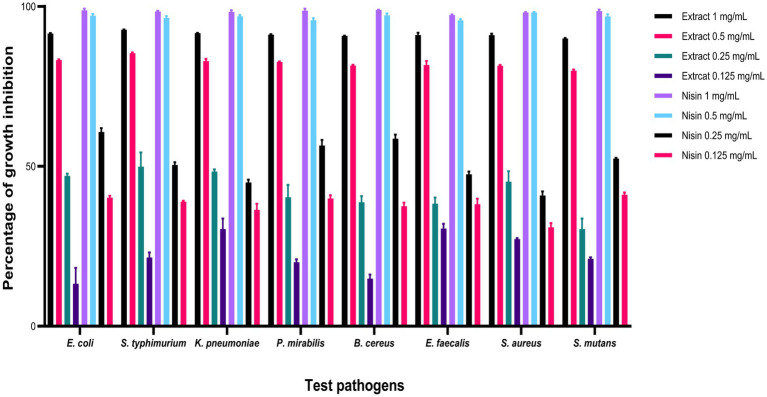
Minimum inhibitory concentration of the probiotic protein extract showing percentage inhibition towards various foodborne pathogens determined by broth microdilution assay. Experiments were carried out in triplicate, and data represent the mean ± SD with *p* < 0.0001.

Minimum inhibitory concentrations of protein extract were determined against different pathogens and compared with standard bacteriocin nisin which served as positive control. Dose dependent effect of protein extract on inhibition of pathogens was observed. 1 mg/mL concentration was found to be effective against all the pathogens showing >90% inhibition followed by 0.5 mg/mL concentration showed 80–90% inhibition. Among all, *S. typhimurium, S. aureus*, *E. coli*, and *E. faecalis* exhibited highest susceptibility. This indicated the broad-spectrum antibacterial activity of protein extract as no major difference was observed between Gram-positive and Gram-negative strains. Substantial decrease in inhibitory effect was observed at 0.25 mg/mL of protein extract resulting in almost half of the bactericidal concentration. It was interesting to note that lowest concentration (0.125 mg/mL) showed varying inhibitory effect unlike higher concentrations where inhibitory effect was uniform among different pathogens. Fair inhibitory effect was found against *K. pneumoniae* and *Enterococcus faecalis* whereas *Escherichia coli* and *Bacillus cereus* were least affected (Figure 2). These observations indicated that the protein extract possesses strong antibacterial effect against different pathogens with its efficacy close to that of purified reference bacteriocin nisin. The efficacy of the protein extract can be increased through various purification steps. MBC (bactericidal concentration) values obtained by spot on lawn method ranged from 0.25 to ≥ 1 as illustrated in Supplementary Figure S2. MIC 50 was determined through non-linear regression dose response analysis, the values ranged from 0.2570 to 0.361 mg/mL (Table 1), demonstrating consistent antimicrobial efficacy across all pathogens (R^2^ > 0.98).

### 3.4 Safety evaluation and stability studies of PPE

**Figure 3:**
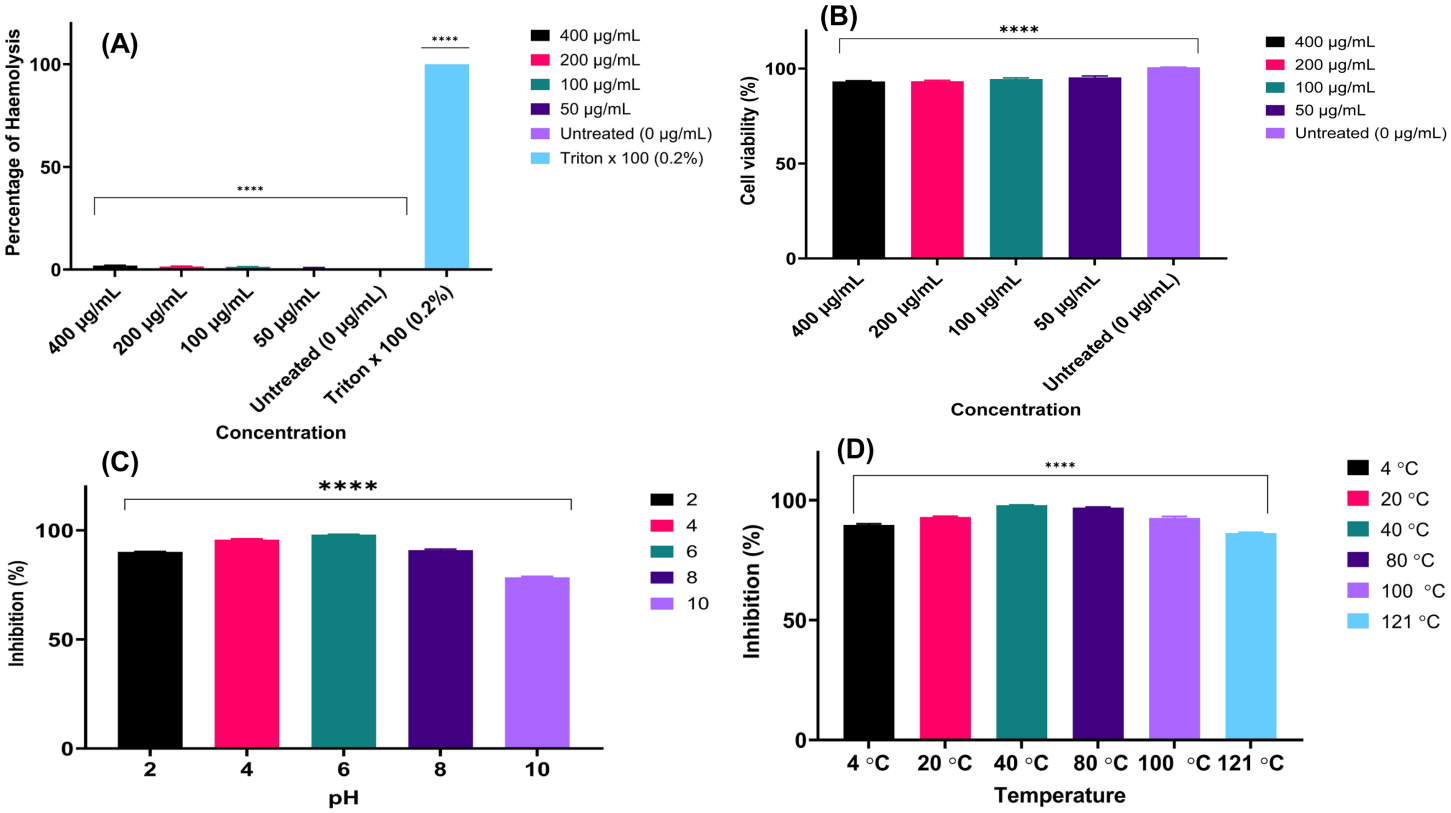
*In vitro* biocompatibility of probiotic protein extract **(A)** Haemolytic activity at various concentrations (μg/mL); **(B)** Effect on cell viability of HEK293 cell lines; **(C)** Effect of pH and **(D)** Temperature on stability.

The ability of extract to show targeted antimicrobial effect on bacterial cells while showing no adverse effects on mammalian cells is very essential safety aspect. To asses this, haemolysis assay was performed and it was found that all tested concentrations of extract were non-haemolytic when compared with positive control (Figure 3A). Further, the protein extract showed no inhibitory effects on viability of HEK293 cell line as evidenced by negligible cytotoxic effect (Figure 3B). The effect of temperature on stability of extract revealed that its inhibitory activity was intact even at higher temperature (121 °C) showing 86.32% inhibition (Figure 3D). The stability of extract at different pH range (2 to 10) revealed its activity to be intact at both acidic and alkaline conditions; however, at pH 8.0 and above, gradual decline in activity was observed (Figure 3C).

### 3.5 Inhibitory effect of PPE on the growth and survival of *salmonella*

**Figure 4:**
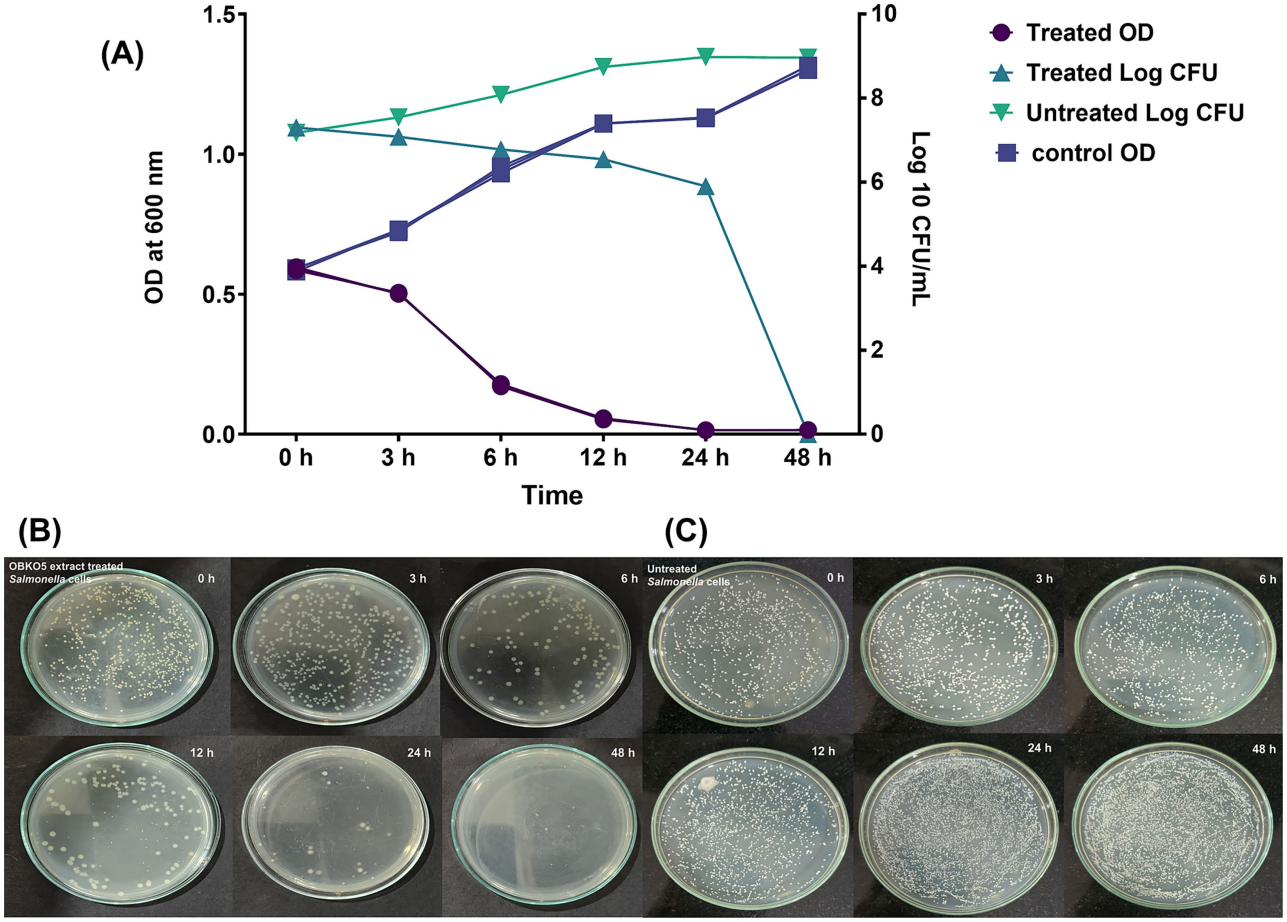
Effect of probiotic protein extract on the growth of *Salmonella* **(A)** graphical representation of growth profile as absorbance and cfu; **(B)** Plate count assay of treated and **(C)** Plate count assay of untreated bacterial culture at corresponding time points. Differences were analysed by one-way ANOVA, and *p* < 0.0001 was considered significant.

The inhibitory effect of the protein extract on the growth kinetics of *S. typhimurium* was monitored over 48 h. The proliferation of bacteria was quantified by measuring absorbance at 600 nm, and the bactericidal efficacy was determined by assessing viable cell counts. At 0 h, both treated and untreated cells showed comparatively similar absorbance values of 0.59 ± 0.006 confirming equivalent cell load at inoculation level. The absorbance of untreated and treated cells at 3 h was found to be 0.73 ± 0.005 and 0.50 ± 0.002, indicating 30.88% reduction in bacterial growth. By the end of 6 h, a significant demarcation was observed where 0.94 ± 0.01 absorbance was obtained for untreated cells and 0.01 ± 0.004 for treated cells corresponding to 81.38% of inhibition. At 12 h, untreated cells showed absorbance of 1.11 ± 0.001 whereas, treated cells measured 0.055 ± 0.001 corresponding to 95.03% growth suppression. The OD_600_ values at 24 and 48 h for untreated cells reached 1.13 ± 0.001 and 1.306 ± 0.008, respectively, while treated cells recorded 0.015 ± 0.008 and 0.015 ± 0.005 of absorbance, corresponding to 98.7 and 98.8% of growth inhibition. These findings were further supported by plate count assay, which showed considerable reduction in the number of viable bacterial cells. The quantitative enumeration (log_10_CFU/mL) revealed bactericidal activity that became apparent by 3 h with a log reduction of 0.46 intensifying to 1.3 by 6 h, 2.2 by 12 h, and 3.07 by 24 h. By the end of 48 h treated cells reached below detection limit representing a log_10_ reduction of 8.96 compared to untreated cells demonstrating complete elimination of the viable pathogens (Figure 4). The considerable decline in both optical density and CFU/mL suggests that the protein extract exerts a potent bactericidal effect by effectively suppressing proliferation of *Salmonella*.

### 3.6 Membrane disruption activity of PPE

**Figure 5:**
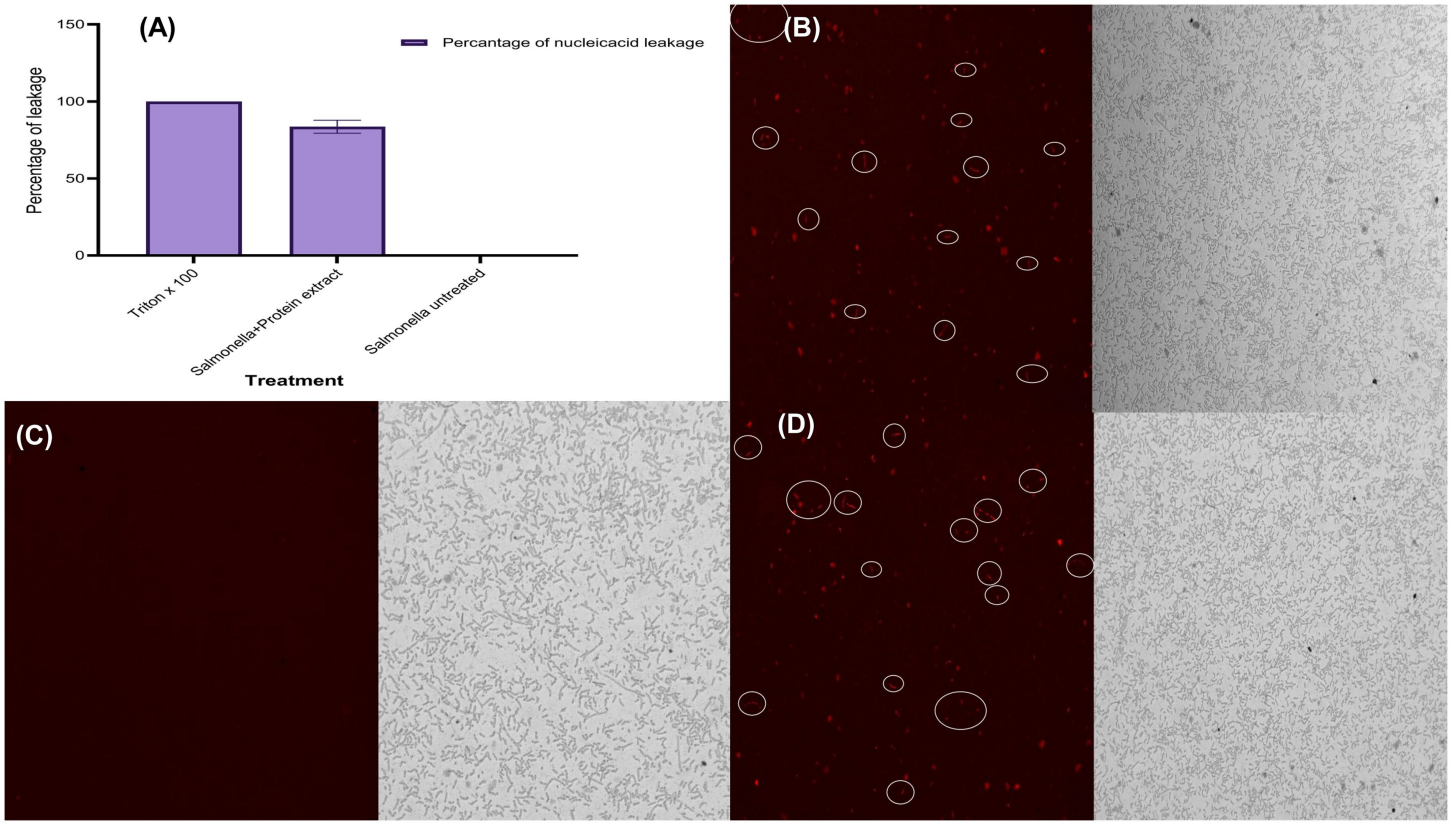
Effect of probiotic protein extract on membrane integrity of *Salmonella* **(A)** Bar graph showing percentage of intracellular component leakage **(B)** Bacterial cells showing red fluorescence by PI uptake after treatment with protein extract; **(C)** Untreated bacterial cells showing no red fluorescence after PI staining; **(D)** Triton X-100 (positive control) treated cells showing red fluorescence in an EVOS live-cell fluorescence microscope with corresponding phase-contrast images for all the treatments.

The extent of membrane damage was evaluated by monitoring the release of extracellular nucleic acids from treated *Salmonella* cells. A significant concentration dependent effect of protein extract on membrane permeability of test pathogen was observed (80% leakage) indicating strong antimicrobial effect. Untreated cells on the other hand exhibited negligible leakage of nucleic acids confirming the intact stability of cell membrane in absence of extract treatment. Positive control (Triton x-100) however showed highest membrane leakage (100%) (Figure 5A). The quantitative leakage of nucleic acids into the medium at a concentration of 500 μg/mL marks major membrane damage, indicating easy permeabilization of extract and irreversible loss of cellular homeostasis; ultimately leading to microbial death.

#### 3.6.1 Propidium iodide uptake assay

The Live cell fluorescence microscopy of treated and untreated cells was observed to determine the extent of protein extract on bacterial cell death.

The fluorescent micrographs (Figures 5B–D) revealed that untreated controls displayed no fluorescence confirming intact membrane structure whereas PPE treated cells showed red fluorescence which indicates uptake of dye. Prolonged incubation led to increased intensity of red colour indicating time dependent uptake of fluorescent dye. Bright filed microscopy confirmed intact cell population but fluorescence imaging displayed prominent red foci within the treated *Salmonella* cells serving as definitive evidence of increased membrane permeability. This suggests that protein extract caused structural damage to *Salmonella* cell envelope enabling dye penetration and disrupting cellular integrity. Collectively, these observations validate membrane disintegration as primary mode of action of probiotic protein extract. These findings were consistent with the morphological changes and biochemical evidence of controlled membrane disruption observed via SEM and nucleic acid release assays.

### 3.7 Impact of PPE on the protein of salmonella

**Figure 6:**
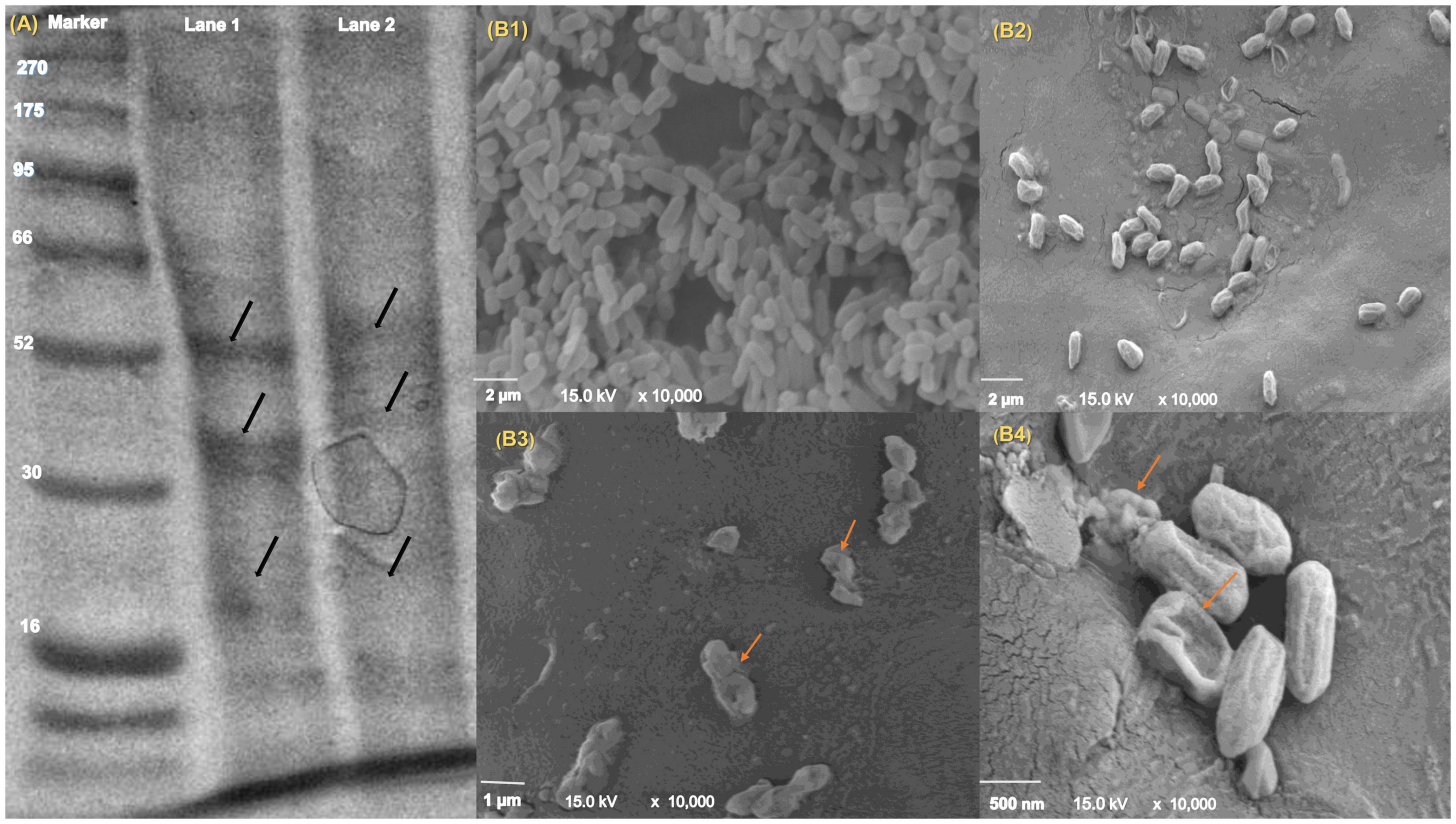
Effect of probiotic protein extract on **(A)** total protein of Salmonella by SDS-PAGE showing Lane 1 with untreated (control) and Lane 2 with protein extract treated Salmonella. Arrows indicate changes in band intensity. The molecular weight marker was run alongside the samples to estimate the size of the protein; **(B)** surface morphology of Salmonella by SEM showing (B1) Untreated *Salmonella* cells with intact morphology (B2) protein extract treated *Salmonella* cells with uneven morphology; (B3) protein extract treated *Salmonella* cells with pore formation, and (B4) with surface distortion.

To assess the impact of extract treatment on bacterial protein, the whole-cell protein was extracted and observed by SDS-PAGE. As shown in Figure 6A, *Salmonella* cells treated with PPE showed a clear change in its band pattern, a new band appeared at approximately 14 KDa and the disappearance of bands at 66 KDa and 40 KDa was observed. However, protein identification was not performed or band intensity was not quantified as these findings represent descriptive changes of the protein profile without mechanistic interpretation. The 14 KDa band may represent protein degradation products or fragments of larger proteins. Further, proteomic analysis such as mass spectrometry is required to determine whether these changes are cellular stress responses or other biological processes.

### 3.8 Morphological changes in *salmonella*

The impact of PPE on the morphology of *Salmonella* cells was visualised by scanning electron microscopy. Untreated bacterial cells showed rod-shaped morphology with smooth cell surface, characteristic of intact cell morphology, whereas the treated cells showed surface deformations and pore formations (Figure 6B). Quantitative morphological analysis revealed a substantial difference in cell surface uniformity between treated and untreated cells. Before treatment, cell surface showed a very low quantitative deviation indicating a uniform intact surface. On the other hand, cells treated with PPE showed increased surface texture heterogeneity with values of 28.21 representing a 3.3-fold increase relative to untreated control. The substantial increase in pixel intensity indicates severe loss of surface uniformity. Additionally, the untreated cells exhibited a preserved membrane integrity with a mean rupture value of 13.33% (ranging from 12 to 16% across individual fields). In contrast, the cells treated with PPE showed mean rupture value of 68.61% (ranging from 53.33 to 90% across replicates) representing a 2.57-fold increase in cell rupture compared to untreated control. A two-tailed, two-sample t-test assuming equal variance was performed where the analysis yielded a *p* < 0.05 confirming that observed difference in treated and untreated groups was statistically significant. Morphological measurements are represented in Supplementary Tables S1–S3; Supplementary Figure S4 as mean ± SD.

## 4 Discussion

Several studies have evaluated the antimicrobial potential of cell-free supernatants derived from probiotic bacteria, which typically contain a diverse mixture of bioactive metabolites, including organic acids, H_2_O_2_, and enzymes (Bhukya and Bhukya, 2021; Lunavath et al., 2023; Barigela and Bhukya, 2021). A critical concern in antimicrobial protein research is validation of protein nature of the bioactive compounds (Bisht et al., 2024). TCA-acetone method is widely followed for crude protein extraction, which precipitates total extracellular proteins. This crude extract may contain non-protein components which gets co-precipitated with protein. To determine whether antimicrobial effect is contributed by protein or non-protein components, catalase mediated decomposition of H_2_O_2_ in the crude extract, was carried out and the results showed antimicrobial activity remained unchanged after catalase treatment, thereby excluding the effect of H_2_O_2_ as major inhibitory factor and this observation aligns with earlier reports of Arena et al (2016). The protease sensitivity assay (Bindu and Lakshmidevi, 2021) was performed to further ensure that inhibitory activity was due to proteins or peptides. The result showed loss of inhibitory activity on pathogenic bacteria after each protease treatment independently is consistent with Ahmad et al. (2014) establishing that the *Pediococcus pentosaceus* OBK05 extract contains protein based antimicrobial activity. The present study primarily focused on protein extraction from probiotic bacteria, and this targeted approach allowed for a more precise assessment of antimicrobial properties of bioactive proteins by separating them from other metabolic byproducts in whole cell-free supernatant (Molina et al., 2025; Heydari et al., 2023; Bindu and Lakshmidevi, 2021). However, the molecular identification and sequential chromatographic purification of the antimicrobial protein remain a necessary step for future research. The probiotic-derived protein extract demonstrated a range of antimicrobial potencies, as evidenced by the agar well diffusion assay. The formation of clear inhibition zones around the wells treated with the protein extract confirmed the presence of bioactive compounds, consistent with previous reports (Tenea, 2020). The difference in the length of the inhibition zone comes from variation in the concentration of active compounds and peptide composition (Upadhyay et al., 2025; Kiani et al., 2025). As noted by Wen et al. (2016), such variations are common in probiotic cultures and significantly influence the overall efficacy of the resulting antimicrobial extract. Recovery of active proteins at the higher concentration validates the efficiency of modified extraction protocol used in the present study and provides a strong foundation for further mechanistic studies (Pessione et al., 2015). The concentrations of crude protein extract and standard nisin were expressed as total protein, and these values serve as a baseline for comparing inhibitory effect but do not reflect difference in specific activity. Future studies will involve isolation and purification of the active protein or peptide and quantify potency in antimicrobial units (AU/mL) to enable accurate comparison with standard bacteriocins. The minimum inhibitory concentration (MIC) assay confirmed a clear dose-dependent antimicrobial effect of protein extract against the test foodborne pathogens. This pattern of growth inhibition was consistent with previous studies, in which increasing peptide concentrations facilitate progressive membrane disruption and ultimately lead to permanent death of bacterial cells (Zhou et al., 2022; Amiri et al., 2022). The vigorous growth inhibition observed at 1 mg/mL that closely aligns with the efficacy reported for other probiotic-derived peptides targeting common food safety threats (Mazumdar et al., 2020). While few synthetic antimicrobial peptides may exhibit lower MIC values (de Moura Cavalheiro et al., 2024), but the antimicrobial efficacy of crude protein extracts can be increased by different purification techniques (Bindu and Lakshmidevi, 2021).

Safety evaluation studies demonstrated negligible haemolytic activity across all the tested protein concentrations compared to the positive control and low cytotoxicity towards HEK293 mammalian cell lines. This indicates, probiotic extract does not compromise RBC membrane integrity and affinity for bacterial cell membrane disruption over eukaryotic cells is a characteristic essential for GRAS status of probiotic derivatives (Liang et al., 2026). The extract showed significant thermal stability which aligns with characteristic properties of heat stable bacteriocin produced by *Lactobacillus paracasie sub* sp. *tolerance* (Miao et al., 2015). Furthermore, pH stability analysis revealed intact antimicrobial activity across the pH range of 2 to 8, where it is advantageous for application in acidic foods.

The growth kinetics and viable counts provide conclusive evidence of strong inhibitory effects by protein extracts isolated from probiotic isolates against *Salmonella* (Pan et al., 2025). A defining feature of this antimicrobial action was the rapid onset of growth attenuation, with a significant deviation from the control observed as early as 6 h post-treatment (Peerzade et al., 2024). This rapid killing effect is a key indicator of the membrane-targeting property of antimicrobial peptides, typically inducing cell death more rapidly than conventional antibiotics that target cell wall synthesis or metabolic pathways (Yasir et al., 2019). While many traditional antibiotics require an incubation period of 12 to 24 h to achieve measurable growth inhibition, the swift response observed in the present study is comparable to the time kill kinetics reported by Zhao et al. (2025) for a highly potent, short-chain bacteriocin. Furthermore, the considerable reduction in viable cell count confirms that the protein extract possesses bactericidal rather than only bacteriostatic properties (Hove et al., 2023). The distinction is critical for food safety applications, as the bactericidal mechanism ensures permanent elimination of pathogens rather than temporary suppression, thereby reducing the risk of microbial regrowth or the development of resistance (Li et al., 2017). It also differentiates these probiotic-derived proteins from other antimicrobial agents that primarily target intracellular biosynthetic pathways, highlighting their potential as rapid membrane disruptors. This controlled permeabilization was important for antimicrobial efficacy while minimising inflammatory responses associated with cell lysis. A critical confirmation of the mechanism of action was demonstrated by detecting extracellular nucleic acids in the supernatants of *Salmonella* treated with probiotic extract. The quantitative release of intracellular components into the medium at a concentration of 500 μg/mL indicates acute membrane damage that extends beyond simple permeabilization, leading to irreversible loss of cellular homeostasis and ultimate microbial death (Yang et al., 2020). This leakage profile was characteristic of potent, lytically active antimicrobial peptides, thereby distinguishing their mode of action from that of agents primarily targeting cell wall synthesis (Chopra et al., 2015).

The membrane-disruption property was further assessed using a propidium iodide uptake assay, which showed time-dependent uptake of the dye, visualised as a progressive increase in red fluorescence intensity over 6 h. These findings support the membrane-disrupting property of the extract, as evidenced by increased permeability and penetration of PI dye into the cell cytoplasm (Rosenberg et al., 2019). While the PI uptake assay confirms cell membrane permeabilisation, it does not distinguish between reversible pore formation and irreversible cell lysis. Future investigations including real-time confocal laser scanning microscopy would enable direct visualisation of membrane depolarisation and pore-formation dynamics at the single-cell level. SDS-PAGE analysis showed changes in the whole-cell protein profile of *Salmonella*, with disappearance or faintness of bands at 66 and 40 kDa and the appearance of a new band at 14 kDa. The appearance of a new band could represent protein degradation or the release of stress proteins in bacteria, and this distinction requires further validation. Previous proteomic studies used advanced mass spectrometry techniques to identify modulation of cellular proteins, including stress proteins and enzymes involved in defence and repair, upon exposure to antimicrobial agents (He et al., 2025; Xue et al., 2016). In our study, such mechanistic interpretation remains speculative without complementary proteomic analysis by mass spectrometry. The rapid onset of this severe damage suggests the probiotic proteins are efficient at inducing bacterial lysis. Alterations in whole-cell protein profiles further reflect the significant proteomic stress induced by exposure to probiotic protein. Scanning Electron Microscopy provided direct visual confirmation of destructive morphological alterations (Sosiangdi et al., 2023) induced in *Salmonella* cells by treatment with the protein extract of OBK05. While untreated control cells maintained smooth, intact surfaces characteristic of healthy, viable bacterial physiology. Conventional SEM analysis relies only on qualitative visual assessment of cell morphology (Alam et al., 2020). In the present study, quantitative image analysis was performed to measure morphological changes, with statistical validation. This provides strong ultrastructural evidence to support a membrane-targeted mechanism of protein extract. The membrane disruption pattern is indicative of protein-induced cell lysis, with the release of intracellular nucleic acids. The observed morphological changes in this study, such as cell shrinkage and pore formation, are highly consistent with mechanisms reported for pore-forming antimicrobial peptides such as nisin and magainin (Rodríguez et al., 2002). Overall, the morphological data, together with functional assays, demonstrate strong consistency across multiple investigative techniques. This multimodal evidence decisively supports the role of compromised cytoplasmic membrane as the primary mechanism underlying bacterial growth inhibition by the probiotic protein extract (Singh et al., 2025). These integrated findings are critical, as they confirm that the bactericidal action is rooted in membrane destabilisation, a characteristic feature of the most successful and non-conventional antimicrobial peptides (Teame et al., 2020). The combination of ultrastructural and biochemical data provides strong confidence that the mechanism of action is lytic and membrane-driven, a mechanism that often exhibits a lower propensity for resistance development than traditional antibiotics (Moravej et al., 2018). In conclusion, this study demonstrates that protein extract from the probiotic isolate *Pediococcus pentosaceus* OBK05 showed potent antimicrobial activity against *Salmonella typhimurium* under controlled laboratory conditions. Collectively, these findings establish a mechanistic foundation for understanding how probiotic-derived antimicrobial proteins interact with bacterial cells at the molecular level. Validating activity in crude or partially purified protein samples is a common step in antimicrobial peptide discovery, ensuring functional confirmation and avoiding expensive downstream analyses (Fugaban et al., 2021). However, it is important to note that these results are only preliminary *in vitro* findings and do not establish practical efficacy in food preservation or therapeutic application. Further translational advancement would require testing in relevant food matrices, evaluation under food processing conditions, and animal studies. Although this study demonstrates strong antimicrobial activity of the probiotic protein extract against laboratory ATCC strains (Leite-Sampaio et al., 2022), the exclusive use of reference strains is a key limitation, as it does not reflect the diversity of clinical or foodborne pathogens. Our future work will test this protein extract against MDR *Salmonella* clinical isolates, ESBL-producing *Enterobacteriaceae* with confirmed resistance genes, and food-derived pathogens from retail/processing environments (Cao et al., 2025). This translational approach will assess the real-world applicability of probiotic-derived peptides as biopreservatives and determine whether the membrane-disruption mechanism confers a lower risk of resistance compared to conventional antibiotics targeting intracellular pathways.

## Data Availability

The original contributions presented in the study are included in the article/Supplementary material, further inquiries can be directed to the corresponding author.
