# Supplementary material for: Bactericidal and cell membrane disruption potential of protein extract from buttermilk-derived probiotic Pediococcus pentosaceus OBK05 against Salmonella typhimurium ATCC 14028
Source: Front Microbiol. 2026 Aug 5;17:1869302. doi: 10.3389/fmicb.2026.1869302 (PMC13485978; doi:10.3389/fmicb.2026.1869302)
Supplement: Supplementary file 1 [file Datasheet1.docx]

**Figure S1.** Minimum bactericidal concentration of Protein extract determined by spot on lawn assay

**Figure S2.** Protease and Catalase sensitivity assay of protein extract by agar well diffusion method (A) Proteinase K treated (B) Pepsin treated (C) Catalase treated

**Figure S3**. Propidium iodide uptake assay left panel- Representative fluorescent (red) images right panel: corresponding phage contrast images of extract treated *Salmonella* cells at 1 h, 2 h, 3 h, 4 h, 5 h, 6 h, Triton x 100 treated Salmonella cells, Untreated Salmonella cells

**Table S1.** Quantitative assessment of cell membrane rupture in Untreated and PPE treated *Salmonella* cells. Values indicate the percentage of ruptured cells across three independent SEM fields. A 2.57-fold mean rupture was observed in treated cells and the difference was statistically significant based on two tailed t-test (*p*=0.034)

| **Image** | **Treatment** | **Rupture count** | **Total cell count** | **Rupture %** |
| --- | --- | --- | --- | --- |
| 1 | Untreated *Salmonella* cells | 3 | 25 | 12.0 |
| 2 | Untreated *Salmonella* cells | 9 | 25 | 36.0 |
| 3 | Untreated *Salmonella* cells | 8 | 25 | 32.0 |
| 1 | PPE Treated *Salmonella* Cells | 8 | 15 | 53.33 |
| 2 | PPE Treated *Salmonella* Cells | 5 | 8 | 62.5 |

**Table S2.** Evaluation of surface roughness using standard deviation of mean intensity. Higher SD represents increased surface heterogeneity and cellular damage. 3.30-fold increase in surface heterogeneity correlates with visual confirmation from 3D topology analysis.

| **Parameter** | **Untreated *Salmonella* cells** | **PPE treated *Salmonella* cells** | **Fold change** |
| --- | --- | --- | --- |
| Mean standard deviation | 8.564 | 28.215 | 3.30 x |
| Surface Uniformity | Uniform | Non uniform | - |
| Cell surface status | Intact | Damaged | - |
| Texture characterization | Homogeneous | Heterogeneous | - |

**Table S3. Quantitative morphological analysis of untreated and PPE treated *Salmonella* cells**

| **Morphological parameter** | **Untreated *Salmonella* cells** | **PPE treated *Salmonella* cells** | **Effect** | ***P-*value** |
| --- | --- | --- | --- | --- |
| Percentage of cell membrane rupture | 26.67 ± 12.0 | 68.61 ± 18.4 | 2.57-fold increase | 0.034 |
| Cell surface roughness (SD) | 8.564 | 28.215 | 3.30-fold increase | Visual confirmation |
| Surface homogeneity | Uniform | Non uniform | Significant damage | - |

**Figure S4.** 3D surface plot showing uniform surface in untreated *Salmonella* cells, larger variation in surface uniformity in treated cells indicating death or damage of the bacterial cells.
